# Extracellular vesicles in the breast cancer brain metastasis: physiological functions and clinical applications

**DOI:** 10.3389/fnhum.2023.1278501

**Published:** 2023-12-04

**Authors:** Yuima Sakamoto, Takahiro Ochiya, Yusuke Yoshioka

**Affiliations:** Department of Molecular and Cellular Medicine, Institute of Medical Science, Tokyo Medical University, Tokyo, Japan

**Keywords:** extracellular vesicles (EVs), breast cancer brain metastases, blood-brain barrier (BBB), biomarker, exosomes

## Abstract

Breast cancer, which exhibits an increasing incidence and high mortality rate among cancers, is predominantly attributed to metastatic malignancies. Brain metastasis, in particular, significantly contributes to the elevated mortality in breast cancer patients. Extracellular vesicles (EVs) are small lipid bilayer vesicles secreted by various cells that contain biomolecules such as nucleic acids and proteins. They deliver these bioactive molecules to recipient cells, thereby regulating signal transduction and protein expression levels. The relationship between breast cancer metastasis and EVs has been extensively investigated. In this review, we focus on the molecular mechanisms by which EVs promote brain metastasis in breast cancer. Additionally, we discuss the potential of EV-associated molecules as therapeutic targets and their relevance as early diagnostic markers for breast cancer brain metastasis.

## Introduction

Breast cancer is the most common cancer among women worldwide and the second leading cause of cancer-related deaths (Goss et al., 2014; Siegel et al., 2020). According to the GLOBOCAN database, in 2020, there were over 2.3 million new cases of breast cancer and 685,000 breast cancer-related deaths in 185 countries (Heer et al., 2020). The number of breast cancer patients is projected to increase further, with an estimated annual increase of over 3 million new cases and over 1 million deaths by 2040, driven by population growth and aging (Arnold et al., 2020). To save more patients, efforts are being made to develop treatment methods, therapeutic drugs, and early diagnostic approaches for breast cancer. While recent cancer immunotherapy has shown clinical success in various types of tumors, it has demonstrated lower response rates in breast cancer compared to other cancers, highlighting the need for further research (Howard et al., 2022). Breast cancer can be classified into several subtypes based on the expression of molecular markers. These markers include HER-2, estrogen receptor (ER), and progesterone receptor (PgR). They serve as therapeutic targets for breast cancer drugs and indicators for selecting effective treatments. Additionally, these molecular markers are crucial for assessing the likelihood of metastasis and disease progression in breast cancer patients (Johnson et al., 2021). For example, triple-negative breast cancer (TNBC) has a higher metastasis rate than luminal-type breast cancer and is associated with a poorer prognosis due to limited effective drugs and the absence of therapeutic target molecules (Kennecke et al., 2010; Zimmer et al., 2022; St-Denis-Bissonnette et al., 2023). These types of breast cancer cells exhibit a high propensity for metastasis to distant organs, particularly the brain and bones (Wu et al., 2017). Among the metastatic sites, the incidence of brain metastasis is higher in breast cancer than in other cancers, with HER-2 positivity and TBNC being the most prevalent (Kuksis et al., 2021). The high occurrence of brain metastasis contributes to increased mortality rates among breast cancer patients. In one study, among 16,703 patients with metastatic breast cancer, 24.6% developed brain metastasis, and the median survival rate after the formation of brain metastasis was 18.9 months for HER2 + /HR + (HR = 0.57, 95% CI: 0.50–0.64), 13.1 months for HER2 + / HR-, 7.1 months for HER2-/HR + , and 4.4 months for TNBC patients, indicating a worse prognosis for TNBC compared to other subtypes (Darlix et al., 2019). Another report showed that the median survival period for breast cancer patients who developed brain metastasis was 45.6 months, while the TNBC group had the shortest survival at 3.5 months (Simsek et al., 2022). These statistical results demonstrate that the frequency of metastasis impacts survival rates.

Brain metastasis often leads to delayed detection and a poor prognosis, especially when central nervous system symptoms are absent. In TNBC patients, the formation of brain metastases occurs in a relatively short time, and the delay in early diagnosis is considered a contributing factor to the higher mortality rate compared to other subtypes (Simsek et al., 2022). Furthermore, the unique property of the brain, protected by the blood-brain barrier (BBB), limits the availability of safe and effective treatment options. The poor prognosis of brain metastasis patients, characterized by high mortality rate and the difficulty of treatment, is speculated to be a significant factor contributing to increased psychological burden on patients and potentially leading to suicidal ideation (Mofatteh et al., 2023). For breast cancer to metastasize to distant tissues such as the brain, circulating tumor cells (CTCs) need to be released from the primary tumor site and adhere to the metastatic site (Bidard et al., 2016; Boral et al., 2017). In recent years, EVs have been reported to play a role in the formation of the tumor microenvironment, the process of cancer metastasis and the formation of metastatic niches (Becker et al., 2016).

EVs are particles separated by lipid bilayers that cannot self-replicate, are released extracellularly from various cells and are present in body fluids such as blood, saliva and tears (Raposo and Stoorvogel, 2013). In 1983, two groups independently described the role of secretory vesicles in the maturation of reticulocytes through the recycling of transferrin and its receptor. At that time, it had not yet been clarified that EVs are responsible for cell-to-cell communication, and these EVs were considered “garbage cans” for disposing of unwanted substances by the cells (Harding and Stahl, 1983; Pan and Johnstone, 1983). Subsequent studies in 1996 reported the presentation of antigens on EVs derived from B cells and their involvement in the activation of T cells (Raposo, 1996), as well as the presence of miRNA in EVs and their transfer between cells (Lotvall and Valadi, 2007), suggesting the potential use of EVs for intercellular information exchange. Secretory vesicles released from cells have different names depending on their size and secretion process, including microvesicles (MVs) with a diameter of 50–1000 nm that are released by shedding from the plasma membrane and exosomes with a diameter of approximately 100 nm that are produced from endosomes (Baixauli et al., 2014). Exosomes are not directly formed from the plasma membrane but are formed inside cells and subsequently secreted into the extracellular space (Raposo and Stoorvogel, 2013).

Exosomes are thought to be formed by budding inward from the cytoplasm into early endosomes, with the involvement of the endosomal sorting complex required for transport (ESCRT) and tetraspanins. ESCRT is involved not only in exosome biogenesis but also in the final stages of MV formation and release (Camussi et al., 2011). The multivesicular bodies (MVBs) that contain numerous exosomes are formed in a shape resembling lipid rafts in the MVB membranes. MVBs can fuse with lysosomes or the cell membrane, and exosomes are secreted only when they fuse with the cell membrane, which is mediated by SNARE proteins (Xu et al., 2022). In contrast, MVs bud directly from the outer cell membrane (Desrochers et al., 2016). The release process of MVs involves the reorganization of cell membrane molecules, including their lipid and protein compositions, and is influenced by calcium levels. Calcium-dependent aminophospholipid translocase and phosphatidylserine promote movement from the inner membrane to the outer membrane, which is considered a typical feature of MVs (Pasquet et al., 1996). The formation and release of MVs are also affected by lipids such as ceramide and cholesterol (Sedgwick and D’Souza-Schorey, 2018). Neutral sphingomyelinase activity, which hydrolyzes sphingomyelin into phosphorylcholine and ceramide, has been shown to be involved in the release of exosomes and the budding of MVs. Inhibition of this enzyme reduces exosome release while increasing MV budding (Menck et al., 2017), suggesting a potential interrelation between the release of these EV subpopulations based on different biological mechanisms. Another type of larger EV is apoptotic bodies, which are secreted by cells undergoing apoptosis and have a diameter of 1–5 μm, larger than other EVs. Apoptotic bodies are characterized by the exposure of phosphatidylserine, which was previously present inside the cell, on the cell surface and their subsequent release (Birge et al., 2016; Battistelli and Falcieri, 2020). Apoptotic bodies also contain fragmented nuclei and cellular organelles and are primarily involved in inducing phagocytosis and subsequent clearance (Zhan et al., 2006). The International Society for Extracellular Vesicles (ISEV) has established the term “EV” as a replacement for “exosome” and “MV” and has established guidelines for distinguishing EV subtypes based on their physical properties. The guidelines set by ISEV 2018 define EV subtypes based on characteristics such as EV size (small EVs < 100 nm or < 200 nm and middle/large EVs > 200 nm), density, and biochemical composition (CD63, CD81, Annexin A5, etc.) (Théry et al., 2018). Tetraspanins (CD9, CD63, and CD81), MVB biogenesis-related proteins (Alix and TSG101), and heat shock proteins are commonly known as proteins specific to small EVs (An et al., 2015; Théry et al., 2018; Kalluri and LeBleu, 2020). EVs carry various substances, including proteins, lipids, and nucleic acids (DNA, mRNA, microRNA, and non-coding RNA), which are expressed by host cells. After uptake by recipient cells, these substances exert various effects and participate in physiological events and the development of diseases related to the maintenance of life in the body (Schiera et al., 2015; Gurunathan et al., 2022). There are numerous reports on the involvement of EVs in cancer, particularly breast cancer (Yi et al., 2022). Cancer cells load their EVs with cancer-specific information such as proteins, lipids, and nucleic acids and deliver it to various surrounding cells (Fujita et al., 2016; Yoshioka et al., 2018; Chen et al., 2019; Giordano et al., 2020), including fibroblasts (Chen et al., 2021) and immune cells (Qi et al., 2022), thereby transmitting the information and promoting the formation of a tumor microenvironment that is advantageous for metastasis and proliferation (Chen et al., 2022).

In this review, we summarize recent research reports on EVs involved in various aspects of breast cancer brain metastasis, such as the formation of the tumor microenvironment and the infiltration of CTCs through the BBB.

## The role of EVs in breast cancer metastasis

**FIGURE 1:**
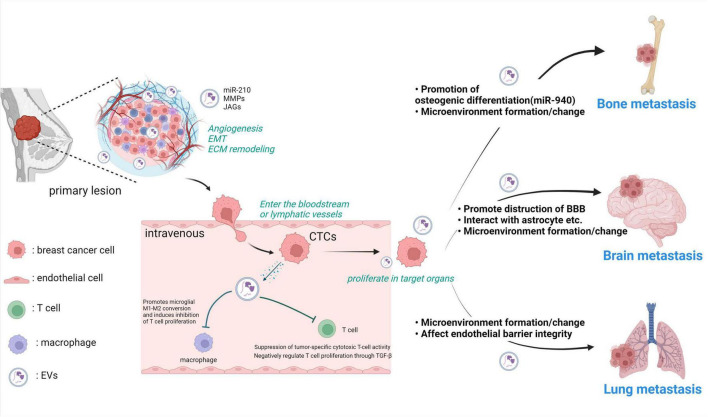
Breast cancer-derived EVs play various roles in the metastatic process from the primary tumor. Breast cancer cells in the primary tumor acquire invasive potential in the microenvironment and locally invade surrounding tissues by reorganizing the ECM. Breast cancer cell-derived EVs alter the microenvironment by internalizing nucleic acids and proteins such as miR-210 and MMPs into the surrounding cells. Breast cancer cells that invade blood vessels become CTCs. They interact with various cells and metastasize to other tissues. Breast cancer cell-derived EVs suppress T cell activity, making CTCs less susceptible to immune clearance. At each stage of metastasis to other organs, metastatic breast cancer cells use EVs to enable metastasis, such as by promoting bone formation and disrupting the barrier function of the BBB. MMP: matrix metalloproteinase, JAG, jagged; EMT, epithelial-mesenchymal transition; ECM, extracellular matrix; CTCs, circulating tumor cells; miR-210, microRNA-210; EVs, extracellular vesicles. Created with BioRender.com.

In the context of metastatic dissemination in breast cancer, the contribution of EV-mediated signaling has been extensively reported across various facets. Metastasis constitutes an intricate biological process wherein primary cancer cells invade adjacent tissues, form neovascular networks, and gain entry into the bloodstream and lymphatic vessels. Subsequently, these cells evade host immune surveillance, disseminate through the bloodstream, and colonize distant target organs, where they proliferate while interacting with the microenvironment of the metastatic site (Fidler, 2003; Brooks et al., 2010). In the subsequent sections, we introduce the association of EVs with each of these processes individually, and the overall is illustrated in Figure 1.

### Angiogenesis

In the initial stages of the metastatic process, cancer growth is enhanced in the primary breast tissue, and the close association of angiogenesis with cancer growth has been well established by numerous studies (Folkman, 1994; Ellis and Fidler, 1995). Angiogenesis not only promotes cancer growth but also plays a crucial role in subsequent metastatic events. In breast cancer, crosstalk between breast cancer cells and vascular endothelial cells, mediated by growth factor secretion, cytokines, and hypoxia induction, is well documented to promote angiogenesis (Li et al., 2001). Additionally, EVs secreted by cancer cells transport angiogenic factors, promoting the formation of new blood vessels by activating vascular endothelial cells (Shi et al., 2022). This concept is supported by numerous reports in breast cancer. For instance, Annexin A2, a Ca^2+^-dependent phospholipid-binding protein that binds to the cell membrane, has been confirmed to be present on the membrane surface of EVs. The expression level of Annexin A2 on EVs was significantly higher in the serum of breast cancer patients than in that of non-cancer patients (Gibbs et al., 2020). In *in vivo* Matrigel plug assays, EVs secreted from TNBC cells were shown to promote angiogenesis through Annexin A2, and this was associated with the worsening of clinical pathological characteristics in breast cancer patients (Maji et al., 2017). Furthermore, it has been reported that neutral sphingomyelinase 2 (nSMase2) controls the secretion of EVs in metastatic breast cancer cells. The EVs released from these cells are rich in angiogenesis-related miRNAs, such as miR-210, which enhances the formation and migratory ability of capillaries by transitioning to endothelial cells (Kosaka et al., 2013). EVs released under hypoxic conditions are also regulated by hypoxia-inducible factor-1α (HIF-1α) induced by low oxygen levels, and these EVs carry miR-210, promoting tube formation in vascular endothelial cells (King et al., 2012). Although hypoxia is known to promote angiogenesis in breast cancer (Semenza, 2016), it is suggested that the increased population of EVs rich in miR-210 and other factors involved in angiogenesis is associated with this low-oxygen-induced process.

### Tissue invasion by cancer cells

The first event in the tissue invasion and metastatic process of cancer cells involves local infiltration of primary cancer cells into the surrounding tissue, achieved through remodeling of the extracellular matrix (ECM) and acquisition of motility (Mohan et al., 2020). During these events, primary epithelial cancer cells undergo multiple biochemical and morphological changes, losing epithelial cell characteristics such as polarity and cell-cell adhesion and transitioning into a mesenchymal phenotype. This process, known as epithelial-mesenchymal transition (EMT), enables the physical dissemination of cancer cells and is influenced not only by intrinsic signaling within the cancer cells but also by various EMT-inducing signals from surrounding stromal cells within the complex tumor microenvironment (TME) (Gonzalez and Medici, 2014). EVs are also implicated in the acquisition of metastatic potential in breast cancer cells. According to Lin et al.’s report, aspartate β-hydroxylase (ASPH) promotes EV secretion in the MDA-MB-231 breast cancer cell line, and these EVs contain factors involved in invasion and metastasis, including active Notch receptors, JAGs, ADAM, and matrix metalloproteinase (MMP), thereby contributing to the promotion of breast cancer cell metastasis (Lin et al., 2019). These EVs enhance the migratory capabilities of breast cancer cells and facilitate infiltration into the endothelium, supporting the invasion of primary tumor cells into the surrounding tissue. Another illustrative example is provided by Singh et al. (2014) who reported that MDA-MB-231 cells infiltrate normal mammary gland cells via EVs. Upon uptake by mammary gland cells, EVs derived from MDA-MB-231 cells suppress the cancer-inhibiting factor miR-10b, thereby enhancing infiltration into the mammary tissue and promoting carcinogenesis (Singh et al., 2014). Breast cancer cells utilize these systems and phenomena to infiltrate the surrounding tissues and progress to the next stage of metastasis. Breast cancer cells in the primary tumor with enhanced proliferation through angiogenesis and dissemination via EMT then invade and spread into blood vessels and lymphatic vessels (Kozłowski et al., 2015).

### Immune evasion of metastatic cancer cells

Intruding breast cancer cells in the bloodstream become CTCs, which approach other tissues through blood vessels or lymphatic vessels. Immune function cells, including macrophages, are present in the bloodstream to eliminate CTCs and other “foreign” cells. Avoidance of immune surveillance is a crucial step in breast cancer metastasis. EVs secreted by the C57BL/6-derived breast cancer cell line E0771 induce apoptosis in recipient cells, such as fibroblasts and stromal cells, suppressing the proliferation of CD8 + and CD4 + T cells and reducing the cytotoxic activity of NK cells against tumor cells in *in vitro* experimental systems (Wen et al., 2016). Furthermore, EVs derived from two different breast cancer cell lines (MDA-MB-231 and BT-474) induced under hypoxic conditions negatively regulate T-cell proliferation via TGF-β and exhibit potent immunosuppressive activity (Rong et al., 2016). Recently, Xing et al. (2018) demonstrated that breast cancer cells with X-inactive-specific transcript (XIST) deficiency, MCF7-shXIST, release EVs containing miR-503, which promote M1-M2 conversion of microglia via the STAT3 and NF-κB pathways in recipient macrophages, inducing inhibition of T-cell proliferation. Indeed, immune regulation of macrophage activity is a critical mechanism employed by breast cancer cells to promote metastasis. EVs secreted by breast cancer cells have been reported to stimulate NF-κB activation in macrophages, leading to the secretion of inflammatory cytokines such as IL-6, tumor necrosis factor-alpha (TNFα), granulocyte colony-stimulating factor (GCSF), and CCL2. Toll-like receptor 2 (TLR2) activation in macrophages is necessary for this effect and is influenced by the presence of palmitoylated protein ligands on the EV surface (Chow et al., 2014).

Interestingly, EVs isolated from breast cancer model mouse tumor cells have been shown to induce the differentiation of bone marrow-derived suppressor cells (MDSCs), which promote tumor progression, via the prostaglandin E2 (PGE2) and TGF-β pathways (Xiang et al., 2009). Additionally, CTCs have the ability to induce platelet aggregation in the bloodstream as a protective mechanism. This not only helps them evade immune surveillance but also facilitates their extravasation and colonization in distant target organs. EVs secreted by highly metastatic MDA-MB-231 breast cancer cells have been shown to induce tissue factor-dependent platelet P-selectin exposure and platelet aggregation (Gomes et al., 2017). Highly malignant breast cancer cells (MDA-MB-231 and 4T1) secrete EVs carrying more TGF-β type II receptor (TβRII). When low-malignancy tumor cells (MCF-7 and 4T07) incorporate these EVs-TβRII, EMT is initiated and metastasis promoted. When EVs-TβRII are taken up by CD8 + T cells, they induce activation of SMAD3, which, in conjunction with the TCF1 transcription factor, exhausts CD8 + T cells and weakens the immune system (Xie et al., 2022). These findings indicate the involvement of EVs in the immune evasion system of metastatic breast cancer cells and suggest that immune evasion events mediated by these EVs may be responsible for the low response rates of breast cancer patients to cancer immunotherapy.

On the other hand, there is a potential to suppress breast cancer metastasis by eliminating EVs that weaken the immune system using anti-EV marker antibodies or similar approaches. Additionally, EVs derived from activated T cells carry PD-1, and they interact with TNBC expressing PD-L1, leading to the internalization of TNBC’s PD-L1 through endocytosis. As a result, it has been reported that this interaction disrupts the PD-L1: PD-1 interaction and reduces the suppression of tumor-specific cytotoxic T cell activity induced by PD-L1 (Qiu et al., 2021). This suggests the possibility of considering treatments that involve engineered EVs with enforced PD-1 expression for metastasis suppression.

## Function of EVs derived from metastatic cancer cells

Many of the CTCs that invade the bloodstream reach distant organs, such as the lungs and liver, through blood flow, causing these locations to become sites of metastasis (Bidard et al., 2016; Boral et al., 2017). Furthermore, at metastatic sites where CTCs arrive, EVs have been shown to play a role in regulating the surrounding environment, enabling a small number of breast cancer cells to proliferate in the target tissue. When monitoring fluorescently labeled breast cancer-derived EVs in live mice, it was observed that they were mostly taken up by lung fibroblasts, enhancing the migration of surrounding cells at metastatic sites and facilitating metastasis (Suetsugu et al., 2013). Reports have also indicated bidirectional interactions between cancer-associated fibroblasts (CAFs) and breast cancer cells through EVs, forming the major cellular components in the TEM. CAFs and CAF-derived EVs showed increased expression of miR-500a-5p, and treating MDA-MB-231 and MCF7 cells with CAF-derived EVs enhanced miR-500a-5p expression in these cells. The increased miR-500a-5p via EVs downregulated ubiquitin-specific peptidase 28 (USP28), which has a role in suppressing cancer cell invasion and EMT. Therefore, the increase in miR-500a-5p promoted breast cancer cell proliferation and metastasis (Chen et al., 2021). Thus, the interaction with surrounding cells through EVs plays an essential role not only in the primary breast tumor site but also in distant organs where CTCs settle by way of the bloodstream.

Breast cancer is characterized by frequent metastasis to the brain and bones (Wu et al., 2017). These tissues are not in close proximity to the primary breast tumor in the breast, and they are considered special metastatic sites because they do not have abundant blood flow, such as that of the lungs and liver, which are common metastatic sites for other cancers. Among breast cancer patients, bone is one of the most frequent distant metastatic sites, with a high frequency of complications and a very poor prognosis due to severe exhaustion (Venetis et al., 2021). CTCs liberated from the primary breast tumor move to the bone marrow through capillaries, acquire bone cell-like properties through osteomimicry, and enhance homing to the bone (Hassan et al., 2012). Adhesion of CTCs to the bone surface is promoted by factors such as the low pH, low oxygen environment in the marrow, and high extracellular calcium concentration (Yang et al., 2019). Once adhered to the bone surface, these CTCs alter the bone microenvironment through proliferation and EV secretion. EVs secreted by bone-metastatic breast cancer cells containing miR-940 promote osteogenic differentiation in human mesenchymal stem cells through the action of miR-940 targeting ARHGAP1 and FAM134A (Hashimoto et al., 2018). Similarly, in a report on prostate cancer, another type of cancer that frequently develops bone metastasis similar to breast cancer, miR-141-3p contained in EVs derived from MDA PCa 2b prostate cancer cells inhibits the expression of DLC1, a Rho GTPase-activating factor, as a target gene, promoting bone metastasis (Ye et al., 2017). The mechanism of bone metastasis involving changes in the bone microenvironment through EVs may or may not be common among different types of cancer. However, the possibility of a common system that efficiently metastasizes to specific tissues exists, and elucidating such mechanisms could be beneficial in establishing therapeutic approaches for bone metastasis.

## Role of EVs in breast cancer brain metastasis events

**FIGURE 2:**
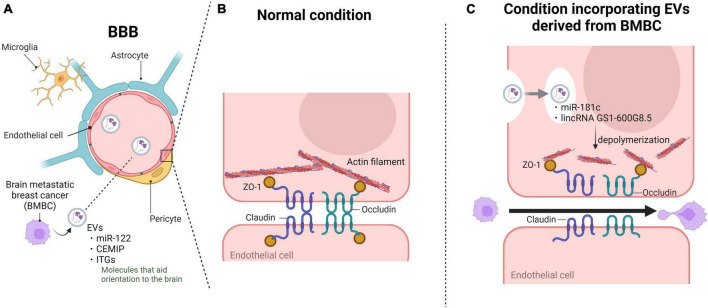
Extracellular vesicles (EVs) derived from brain metastatic breast cancer cells disrupt BBB barrier function and promote cancer cell invasion. Brain metastatic breast cancer cells utilize EVs to disrupt the BBB and facilitate brain infiltration upon microenvironmental formation. **(A)** EVs derived from brain metastatic breast cancer cells circulate in the bloodstream and adhere to brain vascular endothelial cells through proteins conferring target specificity. **(B)** The tight junction complex between vascular endothelial cells primarily consists of tight junctions and adherens junctions, constituting the robust barrier of the BBB. **(C)** Upon endocytosis into vascular endothelial cells, the incorporated EVs destabilize the actin structure and alter the localization of tight junctions, ultimately breaking down the complex and disrupting the BBB. Subsequently, the infiltrated brain metastatic breast cancer cells interact with astrocytes and other components to establish the microenvironment. BBB, blood-brain barrier; CEMIP, cell migration-inducing and hyaluronan-binding protein; ITGs, integrins; ZO-1, cellular distribution pattern of tjp1. Created with BioRender.com.

Breast cancer is the second most frequent cancer to metastasize to the brain, following lung cancer, and the incidence of brain metastasis in breast cancer patients is reported to be between 10 and 30% (Barnholtz-Sloan et al., 2004). The incidence is particularly high in HER-2-positive and triple-negative breast cancer patients (Kennecke et al., 2010; Arvold et al., 2012). For example, in HER-2-positive breast cancer, brain metastasis is more accelerated, with 25–50% of advanced-stage patients being affected (Bendell et al., 2003; Clayton et al., 2004; Yau et al., 2006; Lin et al., 2009; Olson et al., 2013). Brain tumors are more commonly metastases from other tissues than primary brain tumors, and their prognosis is poor, with a high mortality rate due to the lack of effective treatments (Lauko et al., 2020). Although the understanding of the molecules that promote metastasis has advanced, the entry and adaptation of metastasis to the brain are still not fully understood. However, the microenvironment of the brain where CTCs implant is a hostile “soil” for the diffusion of tumor cells, resulting in a low metastatic efficiency. Even in such an environment, breast cancer cells are reported to promote specific metastasis by remodeling the brain microenvironment using secreted EVs to facilitate their own survival and growth (Peinado et al., 2012; Costa-Silva et al., 2015). The main cells that make up the microenvironment of the brain mentioned in this section include neurons, glial cells such as microglia and astrocytes that play a role in maintaining the brain’s internal environment, and endothelial cells that form the BBB and pericytes (Figure 2). Within the group of these cells, the EVs secreted by brain metastatic cancer cells, particularly those targeting glial cells, play a significant role in remodeling the microenvironment. Among these, astrocytes, a type of glial cell, are abundant in the brain’s microenvironment, and their contributions to environmental changes are substantial. Therefore, it has been reported that CTCs interacting with astrocytes play a major role in the formation of brain metastasis. Breast cancer cell-derived EVs with increased expression of the transcription factor tGLI1, which is associated with brain metastasis of breast cancer (Sirkisoon et al., 2020), contain high amounts of miR-1290 and miR-1246. These miRNAs are also detected in circulating EVs in the blood of breast cancer patients with brain metastasis compared to non-brain metastatic patients, even in clinical settings. EVs carrying these miRNAs enhance the secretion of neurotrophic factor (CNTF) by suppressing the expression of the transcription factor FOXA2, thereby activating astrocytes (Sirkisoon et al., 2022). The activation of astrocytes contributes to the progression of brain metastasis in the brain metastatic microenvironment by promoting the proliferation and invasion of brain metastatic breast cancer cells through the secretion of neurotrophic factors such as hepatocyte growth factor (HGF) (Wasilewski et al., 2017). Astrocytes secrete MMP-2 and MMP-9, which promote cancer cell infiltration in brain metastasis of breast cancer (Wang et al., 2013). Regarding MMP regulation, EVs from brain metastatic breast cancer cells are involved in regulating the activity of MMPs. Brain metastatic breast cancer cells secrete small EVs containing miR-301a-3p, which are taken up by astrocytes through a specific Cdc42-dependent clathrin-independent carrier/GPI-anchored protein-enriched compartment (CLIC/GEEC) endocytic pathway. Once these EVs are incorporated into astrocytes, miR-301a-3p inhibits the target gene TIMPs, leading to enhanced MMP activity and facilitating cancer cell infiltration (Morad et al., 2020). Additionally, brain metastatic breast cancer cells interact not only with astrocytes but also with other glial cells. EVs derived from metastatic breast cancer cells induce immune system evasion by microglia through miR-503 (Xing et al., 2018). Moreover, inhibition of glucose uptake in cells within the microenvironment is a characteristic of cancer. Brain metastatic breast cancer cells secrete EVs containing a high amount of miR-122, which, when taken up by non-cancer cells, negatively regulates glucose uptake by downregulating the glycolytic enzyme pyruvate kinase (Fong et al., 2015). In the context of brain metastasis, brain metastatic breast cancer cells, similar to metastasis to other tissues, utilize EVs to modify the microenvironment. Multiple findings presented in this section revealed that astrocytes are the key interacting partners in the microenvironment during this process.

## Disruption of the BBB

The major event in brain metastasis is the breach of the BBB by cancer cells. Due to the lack of classical lymphatic circulation in the central nervous system, CTCs that have detached from breast tissue must overcome the robust barrier of the BBB to reach the brain parenchyma through the bloodstream. The BBB is a highly complex and dynamic structure in the central nervous system that is primarily composed of brain microvascular endothelial cells, pericytes, and astrocytes (Arvanitis et al., 2020). brain microvascular endothelial cells represent the most abundant cellular component of the BBB. Compared to other vascular endothelial cells, brain microvascular endothelial cells express tight junction proteins abundantly and have a very weak endocytic function, which rigorously restricts the entry of various substances into the brain, contributing to the physical barrier between the peripheral circulatory system and the central nervous system (Banks, 2009; Chow and Gu, 2015). In contrast to metastasis to other organs, brain metastasis involves various interactions with the cellular components of the BBB. Cancer cells secrete fluidic factors such as chemokines during this event, leading to the disruption of the BBB and tumor cell extravasation (Li et al., 2017; Curtaz et al., 2020). Similarly, studies have demonstrated that EVs derived from brain metastatic breast cancer cells participate in this process and create a favorable microenvironment for subsequent metastatic cancer cell passage through the BBB. The principal cytoskeletal protein, namely, actin, assumes a significant role by undergoing polymerization to give rise to fibrous structures, thereby governing cellular motility and the establishment of cellular architecture. Furthermore, actin exhibits binding sites for all ZO proteins, including claudin and occludin, thereby being indispensable for the maintenance of tight junctions, which are essential for cellular integrity, on the cellular membrane (Van Itallie et al., 2017; Brunner et al., 2022). To promptly and flexibly address these multifaceted roles, actin systematically disassembles surplus polymerized entities into monomers, engaging in a perpetual oscillation between polymerization and depolymerization states. The disassembly into monomers is chiefly orchestrated by cofilin, and its activity is negatively regulated by 3-phosphoinositide-dependent protein kinase 1 (PDPK1) (Yonezawa et al., 1990). Tominaga et al. (2015) unveiled a novel phenomenon wherein brain metastatic breast cancer cells facilitate cerebral metastasis by attenuating the barrier function of the BBB through EVs. These EVs, originating from brain-tropic breast cancer cells, encapsulate miR-181c. The target gene of this microRNA has been identified as PDPK1. Upon downregulation by miR-181c, PDPK1 activation ensued, thereby stimulating cofilin, a protein that promotes actin depolymerization, facilitating this intricate process. Consequently, modulation of actin dynamics occurred. Furthermore, tight junction proteins, which were originally expressed on the cell membrane, localized to the cytoplasm in cells treated with miR-181c-containing EVs. Although tight junction proteins were not degraded, their expression and localization near the cell wall led to the disruption of tight junction complexes. As a result, the BBB lost its robust barrier function, facilitating brain metastasis of breast cancer cells. Additionally, the level of miR-181c in the serum of breast cancer patients with brain metastasis was significantly increased compared to that in non-brain metastatic patients (Tominaga et al., 2015). These findings have been validated in the serum of patients with established brain metastasis, but they have not yet been confirmed in patients with incomplete or early stages of brain metastasis. However, considering that breast cancer cells with brain metastasis circulate as CTCs and secrete EVs in the bloodstream of patients even in the early stages of brain metastasis, it is expected that miR-181c may be detected at early stages of brain metastasis as well. Lu et al. (2020) established highly brain metastatic breast cancer cells and discovered that they carried an abundance of long non-coding RNA GS1-600G8.5 in their EVs. A comparison between brain microvascular endothelial cells incorporating EVs and brain microvascular endothelial cells incorporating EVs lacking GS1-600G8.5 showed a decrease in the expression of tight junction proteins such as ZO-1, claudin-5, and N-cadherin in the EV-incorporating cells. Although the detailed mechanism by which GS1-600G8.5 reduces tight junction protein expression is unknown. However, it is possible that GS1-600G8.5 may impact the production or stability of these tight junction proteins. These results demonstrated that the disruption of tight junction complexes by EVs promotes increased BBB permeability, facilitating breast cancer cell invasion (Lu et al., 2020). Furthermore, Morad et al. (2019) revealed an alternative mechanism through which brain metastatic breast cancer cell-derived EVs disrupt the BBB without a direct impact on brain vascular endothelial cells (Figure 2). Secreted EVs from brain metastatic breast cancer cells traverse from the vascular side to the brain parenchyma side through endocytic transport within brain microvascular endothelial cells. Subsequently, they are internalized by astrocytes, inducing changes in astrocyte functionality and thereby altering BBB barrier function (Morad et al., 2019). Brain metastatic breast cancer cells break through the robust protective barrier of the brain using these systems, ultimately achieving successful cerebral metastasis.

## Brain-targeted mechanism of EVs derived from brain metastatic breast cancer cells

The uptake of EVs by recipient cells largely depends on uptake systems involving cell membrane receptors and proteins, such as endocytosis and macropinocytosis. The interaction of proteins on EV surfaces with proteins on recipient cell membranes forms ligand-receptor complexes, resulting in differential uptake responses based on the quantity, type, and expression pattern of proteins on EV surfaces, contributing to the directionality of EVs to recipient cells. Organotropic patterns of tumor metastasis have been clinically observed, with lung cancer, breast cancer, and melanoma exhibiting the highest rates of brain metastasis (Suh et al., 2020). Whether these cancer types share common mechanisms that determine organ-specific metastatic patterns is still unclear. However, all of these cancer types are likely to reach the brain through the bloodstream and pass through BBB during the process. It is possible that during this journey, they may influence tight junctions through EVs and other secretions. Recent studies have shown that tumor cells can prerelease EVs that promote their own growth in secondary organs, creating a favorable microenvironment called the premetastatic niche before distant metastasis occurs (Guo et al., 2019). In contrast, for EVs to efficiently reach the secondary organ, which is the site of metastasis, EV organotropism is crucial. Recent research has demonstrated that proteins on EV surfaces play a role in directing organ-specific metastasis. For example, Hoshino et al. in 2015 revealed through proteomic analysis that integrins (ITGs) present on EVs are strongly associated with organotropic metastasis. ITGβ4 and ITGβ3 carried by EVs derived from breast cancer cells specifically mediated lung metastasis and brain metastasis, respectively. Furthermore, this result was confirmed in serum samples from patients with lung metastasis, suggesting that ITGβ4 has the potential to predict lung metastasis in breast cancer patients. However, ITGβ3 did not show specific transport to the brain in patient serum samples (Hoshino et al., 2015). Conversely, Grigoryeva et al. (2023) demonstrated that integrins β3, β4, and αVβ5 expressed on breast cancer cells correlated with brain metastasis. The gap between these findings and clinical samples may be due to differences in the quantity of proteins expressed on EVs and the amount of EVs present. Although not exclusively expressed on the EV surface, molecules that indicate the potential for directionality are exceptions worth mentioning. Rodrigues et al. (2019) discovered that cell migration-inducing and hyaluronan-binding protein (CEMIP), a Wnt-related protein, is highly enriched in EVs derived from brain metastatic breast cancer cells. They demonstrated that CEMIP can promote brain metastasis by increasing the expression of a series of cytokines in microglia, including Ptgs2, TNF, and ccl/cxcl, which are associated with brain metastatic niche formation and colony formation of cancer cells. Interestingly, when CEMIP-knockout breast cancer cells were transplanted into mice, brain metastasis was significantly suppressed compared to non-knockout breast cancer cells, suggesting that CEMIP is involved in determining brain metastasis in breast cancer. The group found correlations between the expression of CEMIP on tissues and EVs and the clinical status and survival of breast cancer patients with brain metastasis, suggesting that CEMIP could serve as a predictive marker for the progression and survival of breast cancer brain metastasis, with CEMIP on EVs potentially being targeted for the prevention and treatment of breast cancer brain metastasis (Rodrigues et al., 2019).

## Glycosylation changes the fate of EVs

**FIGURE 3:**
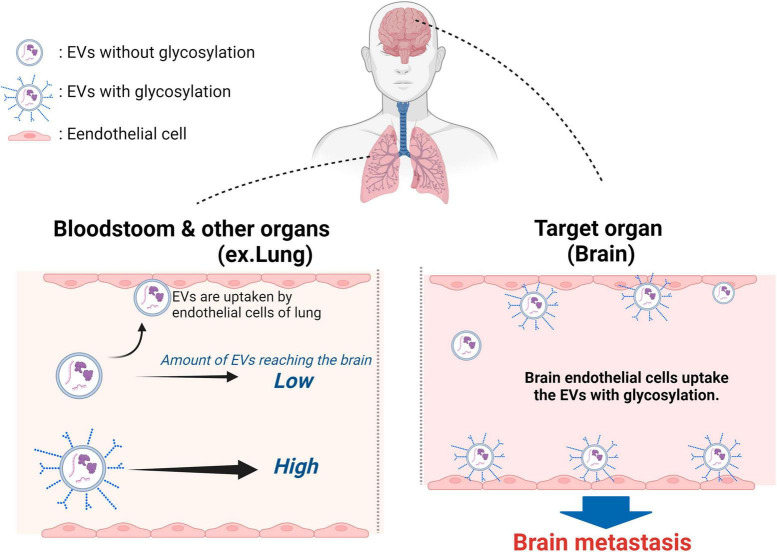
Surface glycans of breast cancer cell-derived EVs have a role in promoting active uptake into brain vascular endothelial cells. The surface glycans on EVs derived from brain metastatic breast cancer cells decrease EV adhesion to vascular endothelial cells in various tissues and tissues near the primary tumor site, thereby increasing the amount of EVs that reach distant tissues. brain metastatic breast cancer cell-derived EVs modified with N-glycans and O-glycans are glycan EVs derived from brain metastatic breast cancer cells modified with N-glycans and O-glycans are not trapped in the inner walls of blood vessels and flow more freely in the blood compared to unmodified EVs. The EVs are then taken up by the vascular endothelium and astrocytes in the brain, which are the target metastatic sites. Many of the EVs derived from brain metastatic breast cancer cells that have undergone deglycosylation become trapped in the lungs. Created with BioRender.com.

Thus far, the focus has been on proteins expressed on EV surfaces as factors determining the tissue selectivity of EVs. However, recent studies have shown that glycans, a major component of EVs, also play a role in EV biosynthesis, cellular recognition, and efficient uptake by recipient cells. Aberrant glycosylation, which is often observed in cancer cells compared to precancerous cells, is known to be associated with cancer progression and metastasis (Mereiter et al., 2019). Furthermore, this abnormal glycosylation affects the glycan profile on EV surfaces, as demonstrated in recent research. Nishida-Aoki et al. (2020) discovered that the glycan profiles of EVs differ depending on the strength of brain metastasis in breast cancer cells. When deglycosylated, EVs derived from breast cancer cells with strong brain metastatic potential exhibited increased uptake by vascular endothelial cells, suggesting that glycan structures on EV surfaces suppress their uptake by endothelial cells. Additionally, O-deglycosylated EVs derived from brain metastatic breast cancer cells significantly promoted accumulation in the lungs compared to non-treated EVs and N-deglycosylated EVs. These findings indicate that glycosylation on the EV surface of brain metastatic breast cancer cells plays a role in avoiding haphazard adhesion to endothelial cells and facilitates the delivery of EVs to the brain, the target tissue. Thus, breast cancer cells that acquire brain metastasis utilize surface glycans to increase the number of EVs reaching the brain, reducing the probability of uptake by organs other than the brain (Nishida-Aoki et al., 2020). This finding suggests that glycans can confer tissue selectivity (Figure 3). Thus, a potential therapeutic target is to suppress metastasis by removing or changing the glycans of EVs that have metastatic potential to the brain or another tissue. Alternatively, the detection of EVs with characteristic glycans suggests that they may function as biomarkers. Additionally, there are examples where cancer cells modify EV surface glycosylation to impart various properties. Cao et al. (2021) demonstrated that N-glycosylation of integrin β1 on EVs derived from highly metastatic triple-negative breast cancer cells (MDA-MB-231) enhances the migratory ability of recipient cells that take up EVs through FAK signaling, promoting metastasis through glycosylated proteins on EVs. Moreover, breast cancer cell-derived MVs carrying highly glycosylated extracellular MMP inducer (EMMPRIN) were found to stimulate cancer cell invasion through the activation of the p38/MAPK signaling pathway in recipient cancer cells (Menck et al., 2015). It has been shown that glycosylation and glycosylated surface proteins of EVs produced by cancer cells are involved in metastasis. Particularly interesting is the finding by Nishida-Aoki et al. (2017) that breast cancer cells with brain metastatic potential may utilize surface glycans to enhance the likelihood of delivering EVs to the target tissue. This functionality may apply to cancer cells that exhibit metastasis to not only the brain but also other distant sites.

## Diagnosis and biomarker utilization of EVs in breast cancer brain metastasis

The early detection of brain metastatic lesions is often challenging. Currently, the commonly employed methods for confirming brain metastasis are imaging examinations and cytological diagnostics. However, imaging studies are not performed frequently, resulting in a time lag until the detection and confirmation of metastasis and tumors, which hinders the accurate reflection of changes (Gupta et al., 2010; Butowski, 2015). Due to the functional and characteristic properties of the brain, brain tissue biopsies can only be conducted during surgery and are not feasible for frequent sampling to monitor tumor progression over time. Additionally, when tumors are located in critical or abnormal brain regions, surgical intervention becomes extremely difficult, with a higher risk of post-operative complications (Patel et al., 2014). Liquid biopsy, in contrast, offers a non-invasive and low-risk alternative with excellent sensitivity, ease of sample collection, and ongoing effectiveness, making it an optimal diagnostic tool for preoperative and post-operative prognoses of breast cancer with suspected brain metastasis. The correlation between the presence of tumors and the quantity of circulating blood-derived EVs has been reported to exhibit a rapid dynamic response. Within 1 day following tumor tissue removal, the levels of tumor-derived EVs in the bloodstream decrease to nearly undetectable levels (Osti et al., 2019; Del Bene et al., 2022), highlighting their high sensitivity. Such swift dynamics, along with the observed correlation between EV quantity and tumor recurrence, suggest a potential role for these changes as biomarkers, both in terms of their rapid kinetics and their potential to provide insights into tumor reoccurrence. As demonstrated thus far, EVs not only effectively reflect the pathophysiology of breast cancer brain metastasis but also carry molecules responsible for the mechanisms of brain metastasis. miRNAs encapsulated within EVs have garnered significant attention in the exploration of biomarkers for predicting and diagnosing breast cancer brain metastasis, with numerous reported correlations (Figueira et al., 2021). EVs derived from brain metastatic breast cancer cells contain a higher abundance of miR-181c than EVs derived from non-brain metastatic breast cancer cells (Tominaga et al., 2015). Furthermore, miR-122, miR-301a-3p, and miR-1290, also found in EVs derived from brain metastatic breast cancer cells, have been reported to be involved in the progression of brain metastasis (Fong et al., 2015; Sirkisoon et al., 2022). However, the validation of these miRNAs and their correlation in actual samples from breast cancer patients with brain metastasis remains a significant challenge, indicating a need for future research to demonstrate their utility. On the other hand, there are miRNAs whose validation has been carried out using clinical specimens. Notably, hsa-miR-576-3p and hsa-miR-130a-3p were assessed in serum-derived EVs obtained from healthy individuals, primary breast cancer patients, and breast cancer patients with brain, bone, or other visceral metastases. The comparative analysis of the expression levels of these miRNAs revealed a significant alteration between patients with brain metastases and healthy individuals. However, the distinctions between breast cancer patients with brain metastases and those with bone or other visceral metastases were marginal and lacked clarity regarding the significance (Curtaz et al., 2022). Changes in miRNA expression are evident not only in EVs derived from brain metastatic breast cancer cells but also in cells within the microenvironment involved in brain metastasis. For example, breast cancer cells that have metastasized to the brain interact with components such as vascular endothelial cells and astrocytes, which constitute the BBB, resulting in changes in their characteristics. Crosstalk between brain microvascular endothelial cells and breast cancer cells results in upregulation of miR-205-5p in breast cancer cells and downregulation of miR-194-5p in brain microvascular endothelial cells. miR-205-5p has been reported to promote EMT (Wang et al., 2016), while miR-194-5p is a tumor suppressor and has been reported to negatively regulate EMT and cancer cell migration (Liu et al., 2020). These miRNAs contained in EVs secreted from each cell can reasonably promote cancer metastasis, suggesting that these miRNAs could be used as diagnostic markers for brain metastasis.

As previously mentioned in the targeting section, EVs derived from brain metastatic breast cancer cells have been indicated to possess surface proteins that confer tropism toward the brain. Once taken up by brain endothelial cells or microglia through EV-mediated transport, these cells express CEMIP, which induces inflammatory cytokines and vascular remodeling, as well as ITGβ3, which is involved in cell adhesion. These molecules are characteristically expressed in brain metastatic breast cancer cells (Hoshino et al., 2015; Rodrigues et al., 2019). Analyzing and diagnosing the miRNA and molecules contained in these EVs, considering complex factors like breast cancer subtypes and individual variations, can be challenging. However, it is possible that algorithms enabling rapid diagnosis and treatment selection could be developed through the utilization of artificial intelligence and machine learning (Li et al., 2023). In addition, they not only serve as biomarkers for brain metastasis risk assessment and progression but also hold potential value in the delivery systems of drugs or molecules to the brain.

## Conclusion

This review discusses the role and impact of EVs in breast cancer brain metastasis. Breast cancer cells adeptly utilize EVs at each stage of brain metastasis events. A unique phenomenon in breast cancer brain metastasis is the incorporation of EVs into the endothelial cells and astrocytes that comprise the BBB, which occurs prior to infiltration to induce BBB dysfunction. This finding suggests a directional delivery of EVs to the intended site of metastasis, and multiple studies have revealed the presence of molecules within EVs that contribute to this targeting ability. These EV-targeting molecules are expected to have clinical applications for breast cancer brain metastasis. Firstly, these EVs can be highly anticipated as early diagnostic markers for breast cancer brain metastasis. However, there are several challenges in utilizing these molecules as therapeutic targets or biomarkers in clinical settings. For instance, the proportion of cancer cell-derived EVs in the circulation is very low, with the majority of EVs originating from other tissues. Therefore, highly sensitive detection methods are necessary to detect cancer cell-derived EVs. As a response to this issue, enclosed miRNAs and nucleic acids within EVs can be detected in very small quantities using amplification methods like PCR. This makes breast cancer brain metastasis-specific miRNAs and nucleic acids within EVs valuable markers. Another issue is the potential for diagnostic inaccuracy due to the influence of breast cancer subtypes and individual variations, which have not been clearly delineated. To address this matter, it is essential to avoid single molecule assessments and instead, combine multiple molecules and nucleic acids.

In addition to directly targeting EV-specific molecules, there is also potential for their indirect utilization in therapy. For instance, their application in drug delivery systems (DDS) is being explored, including the use of artificially created liposomes and EVs derived from mesenchymal stem cells. However, due to the lack of effective local delivery methods to specific target sites, a large quantity of EVs may be required. This raises concerns about the potential for unforeseeable side effects. The molecules and glycans that confer directionality to EVs may offer the prospect of enhancing the localized delivery of therapeutic EVs to the brain and other tissues, reducing the dose of EV administration and mitigating potential side effects.

In the context of targeted treatment involving EVs derived from brain metastasis of breast cancer, the most promising endeavor is the inhibition of brain metastasis. This could be achieved by selectively eliminating or inhibiting breast cancer cell-derived EVs that foster brain metastasis, thereby restraining the disruption of the BBB and impeding the infiltration of CTCs into the brain. The execution of therapeutic strategies targeting these molecules could effectively suppress breast cancer brain metastasis, thereby leading to a decline in breast cancer mortality rates. Given the substantial prevalence of breast cancer patients, the anticipation of significant benefits is well-founded. In fact, there have been reports indicating that the administration of antibodies targeting CD9, which is expressed on the membrane of EVs, to xenograft model mice of breast cancer resulted in the active removal of CD9 antibody-bound EVs by macrophages, consequently leading to the inhibition of lung metastasis (Nishida-Aoki et al., 2017). Other studies have reported cases in which the removal of EVs enhanced the effectiveness of treatment. HER-2 is present on the surface of EVs derived from HER2-positive breast cancer and binds to a therapeutic drug (trastuzumab) that targets HER-2 and consequently inhibits the therapeutic effect (Ciravolo et al., 2012). Marleau et al. (2012) efficiently delivered trastuzumab to cancer cells and inhibited breast cancer progression by removing HER-2-positive EVs from the entire circulatory system using affinity plasma exchange. These observations suggest the potential of EV inhibition for suppressing brain metastasis.

In cancers other than breast cancer, which may potentially metastasize to the brain, it remains unclear whether there are EV-derived molecules showing specificity for targeting the brain. However, EVs derived from lung cancer have been reported to be taken up by vascular endothelial cells, similar to breast cancer, and reduce the expression of tight junction molecules, thereby disrupting the barrier function of brain vascular endothelial cells (Wu et al., 2021). This mechanism is similar to the BBB disruption caused by EVs derived from breast cancer. As a result, the EVs or molecules discussed in this review regarding brain metastasis might find application as promising therapeutic targets. On the other hand, melanoma, which also has a high propensity for brain metastasis like lung cancer, does not have reports of EVs causing BBB disruption. Whether all brain-metastasizing cancers share a common mechanism is a topic for ongoing discussion. If they do have a common mechanism, the molecules on EVs involved in brain metastasis of breast cancer, described within this review, may be applicable as therapeutic targets (Table 1).

**TABLE 1:** The role of EVs in brain metastasis.

EVs cargo/membrane	Role in breast metastatic process	References
miR-122	Reduce glucose uptake non-cancer cells	Fong et al., 2015
miR-181-c	Disrupt the BBB by remodeling actin dynamics	Tominaga et al., 2015
Lnc GS1-600G8.5	Disrupt the BBB by targeting the tight junction proteins	Lu et al., 2020
CEMIP	Promoting b rain metastasis of breast cancer	Rodrigues et al., 2019
miR-1290 miR-1246	Activate Astrocyte through the secretion of CNTF	Sirkisoon et al., 2022
miR-301a-3p	Enhancement of cancer cel l invasion via enhancement of MMP activity	Morad et al., 2020
has-miR-130-3p	cancer-promoting function in connection with RAB5B	Curtaz et al., 2022
miR-503	Induces evasion of immune action by microglia	Xing et al., 2018
Integrins	Promotes adhesion of EVs to target cells	Hoshino et al., 2015

Given the current uncertainty surrounding the effectiveness of surgical removal or chemotherapy for brain metastasis of cancer, it is considered most crucial and effective to suppress the occurrence and progression of breast cancer brain metastasis. Targeting EVs closely associated with cancer activity is expected to be an effective approach to breast cancer brain metastasis. If it is possible to inhibit the secretion of EVs derived from breast cancer, it may not only suppress the proliferation of breast cancer itself but also inhibit further metastasis. However, since the specific mechanisms of cancer EV secretion have not been fully elucidated, the inhibitory effect on EV secretion may also affect other EVs necessary for the body. Therefore, methods that inhibit the trapping of secreted EVs or their uptake by recipient cells are considered the most clinically relevant strategies targeting EVs. Future research on EVs, along with advancements in the collection and analysis methods for EVs, is expected to reveal distinctive proteins and glycans carried by breast cancer brain metastasis-derived EVs to a greater extent. We anticipate that these findings will be applied in clinical practice in the near future.

## Author contributions

YS: Conceptualization, Writing – original draft, Writing – review and editing. TO: Writing – review and editing. YY: Conceptualization, Funding acquisition, Writing – original draft, Writing – review and editing.
